# Evaluating oscillatory mechanisms underlying flexible neural communication in the human brain

**DOI:** 10.1162/NETN.a.550

**Published:** 2026-04-22

**Authors:** Varun Madan Mohan, Thomas F. Varley, Anthony M. Harris, Robin F. H. Cash, Caio Seguin, Andrew Zalesky

**Affiliations:** Department of Biomedical Engineering, Melbourne School of Engineering, University of Melbourne, Melbourne, Australia; The Vermont Complex Systems Center, University of Vermont, Burlington, USA; School of Psychology, The University of Queensland, St. Lucia, Australia; Department of Psychiatry, Melbourne Medical School, University of Melbourne, Melbourne, Australia; Department of Psychological and Brain Sciences, Indiana University, Bloomington, USA

**Keywords:** Communication, Neural oscillations, MEG, Connectome

## Abstract

How the brain orchestrates the flow of information between its multiple functional units flexibly, quickly, and accurately remains a fundamental question in neuroscience. Multiple theories identify neural oscillations as a likely basis for this process. However, a lack of empirical validation of proposed theories, particularly at the whole-brain scale, has hampered consensus on oscillatory principles governing neural communication, limiting our understanding of a process central to perception and cognition and its integration into experiments and clinical applications. Here, we empirically validate previously proposed neural-oscillatory communication mechanisms in the human brain—specifically involving power and inter-areal phase coherence—at the whole-brain scale. We do this by estimating the dependence of inferred communication on oscillatory measures that have been theorized to facilitate communication, in source-localized resting-state magnetoencephalography recordings. We find that power and phase coherence in the alpha, beta, and high-gamma bands track communication better than others. Crucially, the relation between communication and oscillatory measures varied across regions, indicating spatial heterogeneity in routing mechanisms. Notably, power and coherence-based principles tracked communication patterns of unimodal regions better than those of transmodal regions. In sum, these findings suggest that the human brain implements regionally specific communication mechanisms with complex neural-oscillatory dependence.

## Author Summary

Flexible and dynamic routing of information between neural elements is vital for human brain function. Despite its importance, the mechanisms underlying flexible communication in the brain are not fully understood. Several lines of evidence suggest that neural oscillations might mediate flexible communication, but whether such mechanisms operate at whole-brain scales remains uncertain. Here, we investigate the oscillatory underpinnings of communication by assessing how well observed communication patterns from magnetoencephalography data align with the predictions of established theories. Our findings suggest that the brain follows complex region-specific routing principles to realize flexible communication. Knowledge of these principles can develop our fundamental understanding of functions like perception and cognition, refine models of inter-areal interactions, and help develop more effective stimulation-based clinical interventions.

## INTRODUCTION

The brain relies on efficient and effective communication, or routing of information between regions, for healthy function (Avena-Koenigsberger, Misic, & Sporns, 2018; Başar, Başar-Eroglu, Karakaş, & Schürmann, 2001; Buzsáki & Draguhn, 2004; Girardi-Schappo et al., 2021; Griffa et al., 2023; Schipul, Keller, & Just, 2011; Schnitzler & Gross, 2005; Seguin, Jedynak, et al., 2023; Seguin, Tian, & Zalesky, 2020). Neural communication is known to be flexible, or dynamically modulated by several processes, including attention, task/cognitive demands, and processing requirements (Griffa et al., 2017; Lega, Burke, Jacobs, & Kahana, 2016; O’Connor, Fukui, Pinsk, & Kastner, 2002; O’Neill et al., 2015; Voloh & Womelsdorf, 2016), resulting in complex information processing pathways (Avena-Koenigsberger et al., 2019; Cohen & D’Esposito, 2016; Friederici, 2011; Hickok & Poeppel, 2007; Milner & Goodale, 1992). However, despite its fundamental functional role, the information routing mechanism that enables flexible communication in the human brain is not yet fully understood, although several experimental and theoretical advances have identified neural oscillations as a likely basis (Akam & Kullmann, 2010; Engel & Fries, 2010; Engel, Fries, & Singer, 2001; Fries, 2005, 2015; Hillebrand et al., 2016; Odean, Sanayei, & Shadlen, 2023; Osipova, Hermes, & Jensen, 2008; Palmigiano, Geisel, Wolf, & Battaglia, 2017; Reyes, 2003).

Neural oscillation-based theories of communication can be roughly categorised into three broad paradigms—coherence (Fries, 2005, 2009, 2015), power (Buehlmann & Deco, 2008; Jensen & Mazaheri, 2010), or resonance (Hahn, Bujan, Frégnac, Aertsen, & Kumar, 2014)—based on the neural-oscillatory property purported to facilitate communication. Despite their inherent differences, these paradigms may not necessarily be incompatible with each other, as illustrated by works that attempted to unify some of them to arrive at more generalised communication principles (Bonnefond, Kastner, & Jensen, 2017; Hahn, Ponce-Alvarez, Deco, Aertsen, & Kumar, 2019). Several lines of experimental evidence in animal, and to a relatively lesser extent, human studies support oscillatory communication mechanisms (Bosman et al., 2012; Buehlmann & Deco, 2010; Chapeton, Haque, Wittig, Inati, & Zaghloul, 2019; Fries, 2015; von Stein & Sarnthein, 2000; Womelsdorf et al., 2007), although their validity at the whole-brain scale in humans has largely remained speculative.

Notably, the brain is marked by clear heterogeneities in terms of function, oscillatory features, network properties, genetic makeup, directionality of information flow, and so forth, raising the question of whether these heterogeneities extend to routing mechanisms as well. The variation in directionality of information flow across frequency bands at the whole-brain scale (Hillebrand et al., 2016), for instance, suggests such heterogeneity. Brain regions are also regarded to have a complex functional hierarchical organization with implications to communication (Bazinet, Vos de Wael, Hagmann, Bernhardt, & Misic, 2021; Margulies et al., 2016; Mesulam, 1998; Seguin, Razi, & Zalesky, 2019; Vézquez-Rodríguez, Liu, Hagmann, & Misic, 2020). The coarsest level of this hierarchy separates lower-order, functionally specialized unimodal cortices processing sensory information, from higher-order transmodal associational cortices that process multiple diverse streams of information. This differentiation in information flow and processing patterns across the hierarchy could require regions to employ different routing strategies to realize efficient communication. Based on this, we hypothesize that communication in the human brain might be realized through regionally specific routing mechanisms that vary across the cortical surface, and additionally across functional hierarchies, particularly along the unimodal-transmodal axis. Testing these notions would specifically require investigating previously proposed theories in the context of communication at the whole-brain scale.

**Figure 1.**
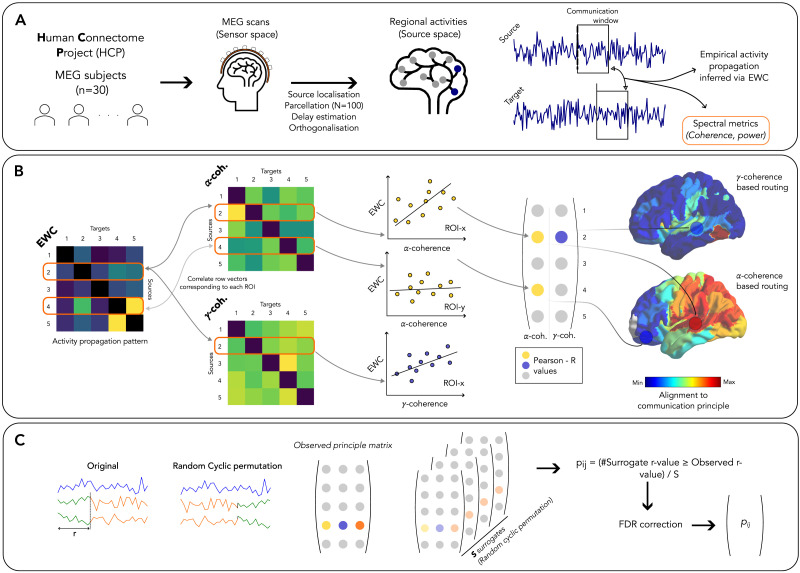
Analyses overview. (A) Preprocessing of magnetoencephalography (MEG) recordings – Resting-state MEG recordings from the Human Connectome Project were source-localized and parcellated. (B) Estimation of regional alignment to routing principles – The communication between regions was systematically inferred using EWC. Within the same windows defined by EWC, neural oscillatory measures were estimated. The oscillatory measures were then correlated to EWC, and the correlation strengths, which captures regional alignment to a routing principle, were projected onto the cortical surface. (C) Surrogate data generation – To minimize biases originating from trivial relationships between EWC and the tested measures, a surrogate dataset was constructed after cyclically permuting target time series relative to the source, prior to carrying out the steps in (B).

In this study, we empirically validate the relationship between inter-regional communication and neural-oscillatory mechanisms at the whole-brain scale by (a) inferring communication between brain regions from neural activity time courses and (b) correlating these to concurrently estimated neural oscillatory measures (Figure 1). We employ a previously developed method to infer inter-areal neural communication, specifically designed to track individual, directional, and high-temporal-resolution signaling events across the human brain, termed Event-marked Windowed Communication (EWC) (Madan Mohan, Varley, Cash, Seguin, & Zalesky, 2025). EWC infers activity propagation from neural recordings using conventional measures of functional connectivity (FC), estimated over select “communication windows” or subsamples marked by salient signal features. This approach enables it to focus on parts of the signal likely to contain instances/effects of inter-areal communication and track effects of both endogenous and exogenous perturbations. Its windowed approach also permits the simultaneous estimation of neural-oscillatory measures within the same communication windows; we specifically focus on the dependence of inter-areal communication on target power and source-target phase coherence (operationalized as inter-site phase clustering [ISPC]).

## RESULTS

Our primary aim in this study was to assess the validity of previously proposed neural-oscillatory mechanisms of flexible communication in the human brain, at the whole-brain scale (Figure 1). We focus on two paradigms of neural-oscillatory communication mechanisms: (a) communication dependent on target power and (b) communication via phase coherence. We used source-localized resting-state MEG recordings of 30 subjects from the Human Connectome Project (HCP) (Figure 1A), from which we inferred inter-areal communication between pairs of brain regions (source and target) and simultaneously estimated target power and ISPC. We restrict our analyses to anatomically connected brain regions, allowing us to focus on how neural oscillations might mediate local signal transmission, which would successively build up the polysynaptic paths between mutually disconnected regions.

We gauge communication using a previously proposed analytical framework called EWC. EWC infers communication between regions by systematically measuring the statistical effects of significant perturbations, or “events,” in the activities of downstream regions. These events are presumed to result in a flow of activity or information from the source, which consequently influences the activities of regions that interact with it. This manifests as an altered statistical dependence, or FC, between the source and target activities, and is quantified to infer the strength of communication between them. Previous work has shown that EWC reliably captures directional communication over network motifs in silico, while also being highly correlated with mainstay inference methods such as transfer entropy (TE) and bivariate Granger causality (GC) when applied to MEG recordings. Additionally, EWC’s windowing approach enables the simultaneous estimation of spectral properties thought to underlie communication and makes it computationally efficient relative to conventional implementations of established methods such as TE (Madan Mohan et al., 2025), further motivating its use in this study (see Methods).

By determining the correlation strength between communication (inferred via EWC) and power/ISPC estimates, we gauged each source brain region’s alignment to a specific communication principle (Figure 1B). These correlation coefficients were then projected onto the cortical surface for visualization, yielding a cortical map of putative mechanisms facilitating regional communication. To ensure that the correlation coefficients did not capture a trivial dependence between inferred communication and oscillatory measures, the maps were filtered using a cyclic surrogate-based threshold (see Methods). By specifying how well the tested oscillatory measures track the information outflow from each source, these maps provide an important step toward the validation of mechanisms of flexible information transfer in the human brain.

### Dependence of Communication on Target Power

**Figure 2.**
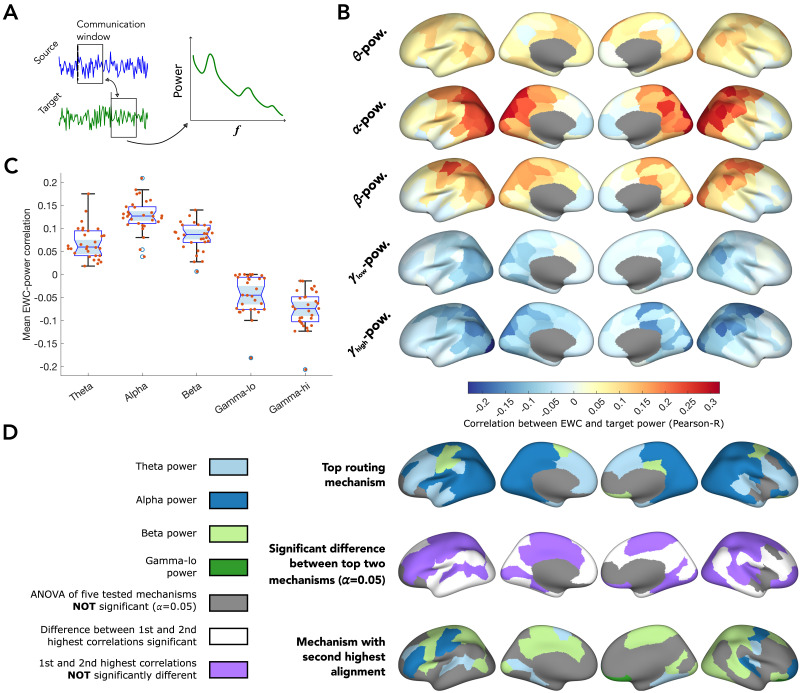
Dependence of communication on target power. (A) For a source and target pair, along with the EWC, the target’s power spectral density (PSD) is estimated for the signal contained in the communication window. (B) The correlation between EWC and target power in each frequency band is projected onto the cortical surface. The colour captures how strongly information flowing out of a region is correlated to target power. (C) Distribution (across all regions) of correlation strengths, to target power in each frequency band (blue circles indicate outliers). (D) (top) Frequency band in which target power maximally correlates with EWC; (middle) binary classification based on whether the top two correlations are significantly different (*α* = 0.05). (bottom) frequency band of the second highest correlation, displayed only in regions where the difference between the top two bands is not significant.

We first set out to characterise the relationship of EWC between pairs of regions (a source and target) to the target’s oscillatory power in the theta, alpha, beta, and gamma (-low and -high) bands (Figure 2A).

We observed a marked spatial heterogeneity in EWC’s dependence with target power in each of the frequency bands, indicative of a location-specific routing mechanism (Figure 2B). Specifically, the dependence of outward information flow on both target alpha and beta power was maximal in the posterior and parietal brain regions typically associated with visual and somatomotor areas, and predominantly positive (Figure 2C). Interestingly, the dependence of inferred communication on both gamma-lo and gamma-hi was also maximal in the posterior regions, but in the negative direction (Figure 2C), indicating that information flow out of those regions were directed to neighbors with low gamma power.

The distribution of the top oscillatory measure (Figure 2D, top), defined as the oscillatory measure with the strongest correlation to a region’s communication, reveals a clear variation of the routing principles along the anterior–posterior axis, with information flow from anterior regions depending maximally on the target theta-power, communication originating from posterior regions depending maximally on alpha-power, and a few regions exhibiting beta power dependence. We then tested whether there was a significant difference between the correlations obtained for the two top mechanisms of each region (see Methods). This step was important to identify regions for which a single oscillatory measure stood out in explaining neural communication inferred using EWC. We did this by first carrying out a one-way analysis of variance (ANOVA) of the correlation coefficients (across recordings) between EWC and the power in each band (*α* = 0.05; in Figure 2D, top, regions for which *p* ≥ 0.05 are grayed out, since there are no “top” routing mechanisms, statistically). For the regions in which the ANOVA indicated a significant difference between the routing mechanisms, we estimated the pairwise differences between group means (each group corresponding to the correlation to power in each band) and assessed whether the top two routing mechanisms were significantly different (*α* = 0.05) (see Statistical Analyses). We found that the correlation of the top routing principle was statistically similar to the second-best routing principle (pairwise comparison of group means *p* ≥ 0.05; beta power in most cases—Figure 2D, middle; regions in violet) in a large number of regions. However, there were also several regions—located predominantly in medial prefrontal, temporal, and occipital areas—whose communication patterns were best explained by target power in a single frequency band (pairwise comparison of group means *p* < 0.05; Figure 2D, middle; regions in white). Notably, we found only one region, the right orbital frontal cortex, where gamma band power, specifically gamma-lo, predicted routing.

In short, our investigation into information routing in the brain based on power reveals a rich heterogeneous landscape of the relationship between communication and target power. We find that the dependence of communication on power varies not only with the frequency but also spatially across the cortical surface. This indicates that signaling originating from different regions might be facilitated by different oscillatory mechanisms. We also find evidence suggesting that a routing principle based on oscillations in a single frequency band might not fully capture empirical communication mechanisms.

### Dependence of Communication on Phase Coherence

**Figure 3.**
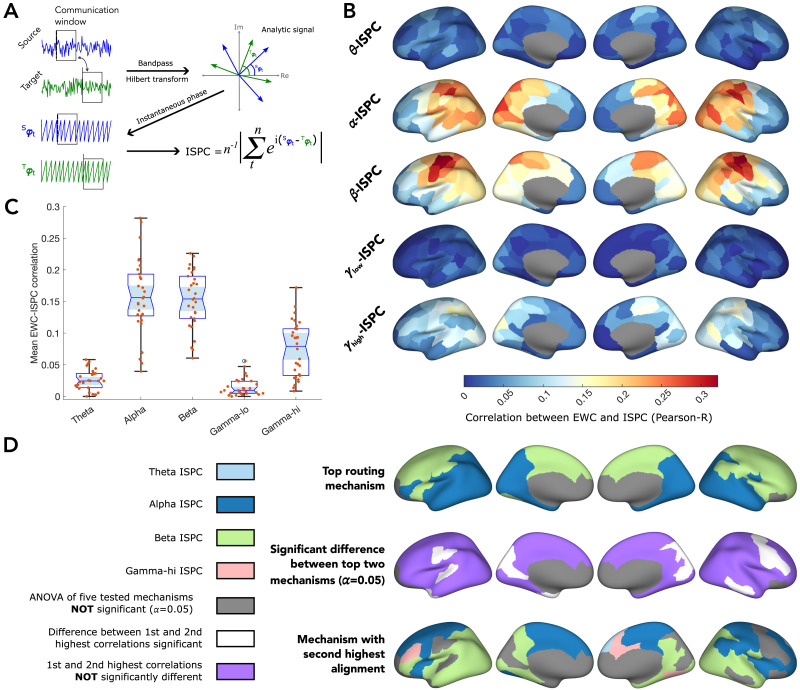
Dependence of communication on coherence. (A) For a source and target pair, the signals are first bandpass filtered into the theta, alpha, beta, gamma-lo, and gamma-hi bands. The phase time series is then extracted from the signals after Hilbert transform. The ISPC is then estimated from the phase time series, along with EWC, which is estimated from the raw signals. (B) The correlation between EWC and ISPC in each frequency band is projected onto the cortical surface. The colour captures how strongly information flowing out of a region is correlated to ISPC. (C) Distribution (across all regions) of correlation strengths, to ISPC in each frequency band. (D) (top) Frequency band in which ISPC maximally correlates with EWC; (middle) Binary classification based on whether the top two correlations are significantly different (*α* = 0.05). (bottom) Frequency band of the second highest correlation, displayed in only those regions where the difference between the top two bands is not significant.

In our second set of analyses, we investigated another paradigm of neural-oscillatory mechanisms—communication through phase coherence. Like the approach in the previous analyses, here, we correlated inferred communication patterns captured by EWC to the ISPC between sources and anatomically connected targets in the five frequency bands, within the same communication windows (Figure 3A): ISPC captures the consistency between phases of a pair of signals, indicating the level of synchronization between them (see Methods). Projecting the correlation values onto the cortical surface, we found that the dependence of communication on ISPC followed a spatially heterogeneous profile as in the case with target power (Figure 3B). For instance, while both alpha and beta ISPC showed similar correlation to EWC across regions (Figure 3C), the regions where alpha coherence strongly correlated with communication were primarily localized in the posterior part of the brain, whereas beta coherence correlated maximally with EWC in parietal and somatomotor regions. Across regions, ISPC in the alpha, beta, and gamma-hi bands correlated with EWC better than theta and gamma-lo bands (Figure 3C).

As in our analyses with target power, for each region, we assessed the significance of the difference between the correlation of EWC to the ISPC in different bands via a one-way ANOVA, identified the top two bands in which ISPC maximally correlated with EWC, and tested whether the correlations were significantly different. As argued previously, this was to ascertain whether only a single oscillatory measure could predict a region’s communication with its neighbors. Although looking at measures with the maximal correlation to EWC clearly revealed alpha ISPC dominance in posterior inferior regions and beta ISPC dominance in the anterior regions (Figure 3D, top), we found that in most regions, the second highest correlation was statistically similar to the first (pairwise comparison of group means *p* ≥ 0.05; Figure 3D, middle; regions in violet), suggesting that ISPC in at least two bands similarly tracked EWC patterns. Regions in which ISPC in a single band tracked EWC better than others were much fewer (pairwise comparison of group means *p* < 0.05; Figure 3D, middle; regions in white), located in the medial occipital, left superior temporal and central, and right inferior temporal and frontal areas. It was further interesting to observe that in most regions where the maximal EWC correlation was to alpha ISPC, the second highest correlation was to beta ISPC, and vice versa (Figure 3D, bottom).

To summarize, in this set of analyses, we investigated the degree to which whole-brain communication aligned with coherence-based information routing principles. We found that communication, as captured by EWC, had a notably heterogeneous dependence on coherence in the different bands along the cortical surface, as evidenced by the non-uniform correlation strengths between the measures across regions. Additionally, the cortical distribution also varied with the frequency band, indicating regional specificity in the routing principle followed. ISPC in the alpha and beta bands correlated the most strongly with EWC (pairwise comparison against theta, gamma-lo, and gamma-hi bands; *p* < 0.05). We further found that in most brain regions, ISPC in at least two frequency bands could similarly predict information flow captured by EWC.

To ensure that the heterogenous dependence of EWC on power and coherence was not dataset-specific, arising from features such as acquisitional parameters or sampling rates (SRs), or due to our choice of parcellation atlas, we carried out similar analyses on a separate dataset, that differed in all these features (Supporting Information Figure S2). Relying on a qualitative/visual comparison of the EWC-power/ISPC correlation distributions due to the different parcellations, we noted that there were several similarities in spatial distribution of EWC-power/ISPC correlations between the datasets (Figures 2B and 3B, Supporting Information Figure S2). For instance, the dependence of EWC on alpha power was higher in posterior and parietal regions, the dependence on beta power was higher in parietal regions, and the dependence on gamma-hi power was also dominant in posterior regions, but in the negative direction. Similarly, in the case of EWC’s dependence on ISPC, results from both datasets showed communication of posterior regions to have a higher alpha band ISPC dependence whereas dependence in the beta band was maximal in parietal regions.

### Neural Oscillatory Dependence of Communication Across Functional Gradients

**Figure 4.**
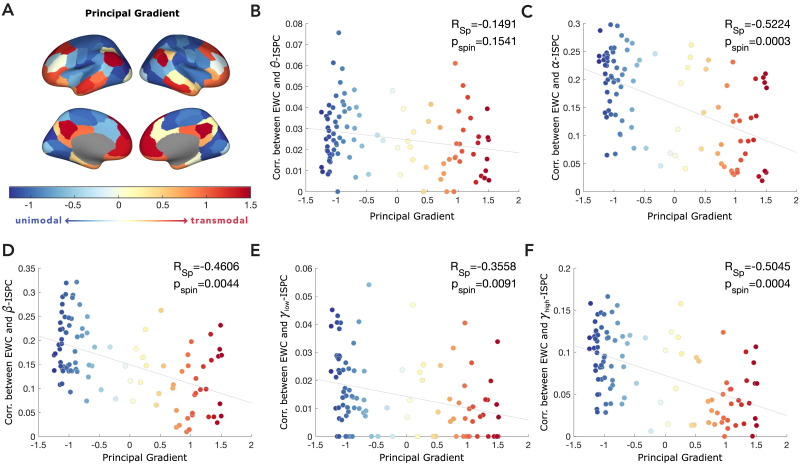
Strength of the correlation between a EWC and ISPC depends on the source region’s placement along the principal functional gradient. (A) Principal functional gradient derived from diffusion embedding of group-averaged FC. (B–F) Correlation between EWC and ISPC in the frequency bands of interest, as a function of the source region’s principal gradient coefficient (also represented in the colour of the point). Each point represents a brain region. Relationship to functional gradient is quantified as the Spearman correlation, and *p* value is obtained by comparison to surrogate dataset with 10^4^ spin permutations.

Our previous analyses revealed clear spatial and spectral heterogeneity of inferred communication’s dependence on power and ISPC. The parietal and posterior localization of the regions showing maximal correlation to the oscillatory measures, however, prompted us to explore whether the extent to which these measures tracked communication varied with gradients in functional organization, particularly about the unimodal-transmodal axis (Margulies et al., 2016; Mesulam, 1998). In our final set of analyses, we estimated the degree of spatial alignment between the EWC-ISPC correlation maps obtained in our previous analyses and the principal functional gradient derived from group-level FC maps (Figure 4).

We also estimated the gradient’s alignment to the EWC-power maps (Supporting Information Figure S3). We observed that the correlation strength between inferred communication and coherence varied across the principal functional gradient delineating the unimodal-transmodal axis, for the alpha (Spearman *R, R*_*sp*_ = −0.5224, spin permutation-based *p*_*spin*_ = 0.0003), beta (*R*_*sp*_ = −0.4606, *p*_*spin*_ = 0.0044), gamma-lo (*R*_*sp*_ = −0.3558, *p*_*spin*_ = 0.0091) and gamma-hi (*R*_*sp*_ = −0.5045, *p*_*spin*_ = 0.0004) bands. Interestingly, the variation across the gradients for all bands revealed that the correlation between ISPC and communication was stronger for unimodal regions compared to transmodal regions. This difference was most prominent for the alpha band, whereas the dependence of communication on theta ISPC did not show significant variation across the unimodal-transmodal axis. Like ISPC, power also tracked communication primarily in unimodal regions (Supporting Information Figure S3).

To summarize, in this section, we examined the utility of ISPC and power in explaining a region’s communication patterns given its position in the functional unimodal-transmodal axis. We found evidence suggesting that communication based on both phase coherence as well as power, particularly in the alpha, beta, and high gamma bands is more dominant in unimodal regions rather than transmodal regions.

## DISCUSSION

The mechanisms and principles underlying communication over different scales, that enable the brain to perceive, process, and act on stimuli within milliseconds, have understandably been a topic of much interest (Avena-Koenigsberger et al., 2018; Lerner, Ye, & Deisseroth, 2016; Roland, Hilgetag, & Deco, 2014; Seguin, Sporns, & Zalesky, 2023). Over the years, there has been an accumulation of a wealth of experimental data, insights and theories of neural communication, and the development and refinement of biophysically accurate models, allowing us to develop means to observe and study brain communication in great detail. Although neural oscillations are widely considered to underpin mechanisms of flexible communication, there is a lack of consensus on the exact mechanism that operates in the human brain and whether the oscillatory dependence is consistent across regions. In this work, we set out to address this outstanding question and concurrently place established communication theories in the more general context of information transfer at the whole-brain scale.

We specifically explore two flexible oscillatory communication paradigms: communication dependent on target power and communication through phase coherence. To do this, we first inferred the communication between anatomically connected regions and simultaneously estimated oscillatory measures associated with proposed theories. This was followed by estimating the correlation between them, effectively capturing the alignment of regional communication toward putative routing mechanisms. Across both the tested paradigms, alpha and beta band oscillations were the most associated with communication (Figures 2C and 3C). In the case of EWC-power correlations, it could be argued that the observations result from inherent power heterogeneities and non-uniform spectral densities. However, the same cannot be said for EWC-ISPC correlations since the ISPC is estimated on the instantaneous phase time series of a band-passed signal and effectively power normalized (Bruña, Maestú, & Pereda, 2018; Cohen, 2014). Despite this, it was noteworthy that the observed heterogeneities in ISPC dependence seemed to match inherent regional power heterogeneities, particularly in the alpha (posterior dominant) and beta (dominant in parietal somatosensory areas) bands. This suggests that regional power distributions may play a role in determining what oscillation-based routing mechanism is followed by a region. Additionally, it is interesting to observe that in both the paradigms, in most regions, the top two oscillatory measures track EWC in a statistically similar manner (Figures 2D and 3D). This suggests that empirical communication mechanisms might involve multiple frequency bands, as opposed to communication over a single band. A routing mechanism based on the observed heterogeneous oscillatory dependences would imply that regions locally communicate via the same frequency band, but over different frequency bands for distant targets, in a manner similar to the distance-based routing mechanism earlier proposed by von Stein and Sarnthein (2000).

In our analyses of the dependence of inferred communication (via EWC) on target power, it was interesting to observe the positive association of EWC to target alpha power and the negative association to target gamma power. At first glance, these observations seemed to be in contradiction to literature linking alpha to functional inhibition and gamma activity to neural processing, which would suggest that the associations be sign-flipped (Jensen & Mazaheri, 2010). A better interpretation of our observations requires a closer look into EWC and what it measures: the lagged partial correlation between source and target signals, essentially capturing the degree of synchronization between the signals in the time domain. From a signal processing standpoint, an increase in the high-frequency gamma band component of the target would imply stronger rapid fluctuations of the signal and might serve to reduce synchronization, effectively resulting in a negative association. On the other hand, an increase in inhibition, which is well associated to increases in alpha power, is also counterintuitively linked with an increase in synchronization (Hanslmayr, Staudigl, & Fellner, 2012; Klimesch, Sauseng, & Hanslmayr, 2007; Van Vreeswijk, Abbott, & Ermentrout, 1994), which might explain the positive association between EWC and target alpha power. Future work combining neurotransmitter imaging with localized neural recordings, or using a measure for differential neural processing capabilities between pairs of regions as a proxy for communication will be able to establish this link more definitively. Our seemingly counterintuitive observations also help remind us that the low-level signal features we observe do not always map directly to cognitive/behavioral implications, which often require several additional levels of insights and inference.

Our results also revealed that communication in the anterior regions, often associated with higher-order brain function, showed negligible oscillatory dependence in the case of power, and only a weakly positive dependence with ISPC in the alpha, beta, and gamma-hi bands (Figures 2B and 3B). This was further emphasized in the functional gradient analysis, where the coherence and power dependence in transmodal regions were found to be lower than in unimodal regions (Figure 4). This could suggest that higher-order regions might follow a more complex oscillatory, or perhaps even non-oscillatory, routing mechanism, to facilitate the integration of information from various input streams.

Testing the oscillatory dependence of communication in an empirical setting is advantageous on many fronts. For instance, knowledge of the principles describing communication dynamics will help us better understand how the underlying structural connectivity (SC) relates to observed FC (Avena-Koenigsberger et al., 2018). On the computational modeling front, the incorporation of flexible communication will make models of whole-brain activity more realistic. It can also help build intuition into communication dynamics associated with neural oscillatory changes linked to cognitive task processing and neuropathologies. In a clinical setting, understanding the principles of communication dynamics can help in predicting and effectively controlling information flow through stimulation-based interventions such as transcranial magnetic stimulation or deep brain stimulation. By capturing the regional variations in the alignment of communication to different frequency bands, our results provide empirical support for existing communication theories involving various oscillatory measures and frequencies (Bonnefond et al., 2017; Buehlmann & Deco, 2010; Chapeton et al., 2019; Engel & Fries, 2010; Fries, 2005, 2015; Jensen & Mazaheri, 2010; Palmigiano et al., 2017) in a whole-brain context, while also identifying potential limitations to their validity. A future direction of research could explore how we can combine all the correlation strength maps to arrive at a comprehensive communication principle for the cortical surface. One possible model would be a winner-takes-all approach, where the communication principle of a region is defined as the spectral metric that correlates the most with communication. The most important assumption there would be that the other spectral metrics might not facilitate communication, or have negligible contributions compared to the metric that is the most correlated with communication strengths. A model that would not discount the contributions of the other bands might be one in which the routing principle is the sum of the various spectral metrics weighted in proportion to their location specific correlation strengths. Furthermore, although we only explore the dependence of communication on power and phase coherence in this work, future work could also test the validity of other oscillation-based theories such as communication through resonance (Hahn et al., 2014), or those simultaneously involving multiple frequency bands and measures (Bonnefond et al., 2017; Hahn et al., 2019). We also note that future work could apply our EWC framework to study the time-resolved employment of different oscillation-based mechanisms of neural communication. This could reveal whether different brain regions—with distinct structural and functional profiles—dynamically recruit and switch between modes of neural communication over time.

Finally, an alternative approach to conceptualizing how information is routed between regions is through network-based models, where the white matter, or SC between regions, determines the route and efficacy of information transmission between them. These models have been shown to adequately predict function and even stimulus propagation paths (Seguin, Jedynak, et al., 2023; Seguin, Sporns, & Zalesky, 2023; Seguin et al., 2020). Flexible information flow can also be realized through a dynamic anatomical substrate (Pope, Seguin, Varley, Faskowitz, & Sporns, 2023). The network-based and oscillation-based paradigms are not mutually exclusive, but rather capture different aspects of the routing process. While network-based models describe possible communication routes between regions for a given network, oscillation-based models describe the mechanism by which those routes may be realized. Bridging these paradigms, by exploring oscillation-based routing principles as a generative model for network-derived communication routes, could be an important direction for future research.

### Limitations

While our results suggest that information transfer in the human brain follows heterogenous routing principles, it is essential to consider the analytical and methodological limitations of this study to better contextualize our findings. Firstly, it is important to mention that our notion of information transfer/communication is statistically inferred from neural activity time courses—it is not a direct measure of regional responses to a perturbation, as in the case of localized neurostimulation-response measurements. Secondly, the employed inference framework (EWC) operates under several key assumptions. For instance, the operational parameters such as the window length, Euclidean distance-proportional delays, and event detection *z*-score threshold were fixed based on insights from previous work and literature, in a cohort of healthy individuals, to adequately capture the downstream effect of events, while containing enough data points for reliable estimates and spectral analyses. These parameters, however, are not optimized for each recording. The *z*-scoring approach can also be affected by drifting means within an epoch or high frequency spiking for long durations, which can inflate the mean and equivalently result in the spikes not being identified as salient events and instead as a “new normal” for the epoch. Additionally, the measure we use to gauge the statistical dependence in EWC, the partial correlation, although simple and extensively used in neuroscience, assumes linearity of the underlying dynamics. Therefore, partial correlation, and consequently our measure of communication estimated via EWC, will only reflect linear dependencies. A nonlinear version would see the use of measures such as the conditional mutual information estimated using a binning or nearest-neighbor approach. Thirdly, the need for non-invasive whole-brain subsecond resolution neural dynamics drove our choice for source-localized MEG to study the principles of endogenous communication. Although source localization yields region of interest (ROI)-level recordings, the reduced spatial resolution of MEG can cause sensor-level signals to be “smeared” across sources, and subject to assumptions of the source localization technique used. If the spatial extent of the investigation is reduced, more spatially localized modalities such as intracranial EEG would help bypass several such limitations. Finally, it is crucial to acknowledge that although we had to infer communication as well as estimate the neural oscillatory measures from the same underlying signal for the current study, requiring us to employ a surrogate-based correction of observed relationships, an ideal approach would be to estimate these measures separately from concurrent and independent modalities when available.

### Conclusion

In conclusion, our work explores the validity of neural oscillation-based mechanisms of communication at the whole-brain scale. We find that measures like power and phase coherence, theorized to facilitate communication processes, track communication in a regionally specific and hierarchical manner. Our findings shed light on the complex neural oscillatory underpinnings of communication in the brain.

## METHODS

### Dataset

#### Human Connectome Project (HCP).

Resting-state MEG scans of 30 subjects (22–35 years, 17 females), along with associated MEG anatomical data, 3 T structural MRI data, and empty-room recordings, were obtained from the HCP (Van Essen et al., 2013), through the ConnectomeDB platform. MEG was acquired using a 4D Neuroimaging MAGNES 3600 system at an SR of 2035 Hz (Larson-Prior et al., 2013). Recordings varied in duration from 5–6 min, and anti-aliasing low-pass filtered at 400 Hz.

#### Open MEG Archive (OMEGA).

To test the generality of our findings beyond one dataset and set of acquisition parameters, we carry out all our analyses on a second dataset—a subset of the OMEGA dataset. Resting-state MEG scans of five subjects (21–35 years, 2 females), along with 3 T structural MRI data, and empty-room recordings collected as a part of the OMEGA initiative, was obtained from OpenNeuro (dataset version 1.0.2: https://dx.doi.org/10.18112/openneuro.ds000247.v1.0.2; Niso et al., 2016). The recordings were acquired using a CTF 275 system with an SR of 2,400 Hz. Recordings were 5 min long, and anti-aliasing low pass filtered at 600 Hz.

### Processing

The MEG recordings from both HCP and OMEGA datasets were processed entirely using the Brainstorm software (Tadel, Baillet, Mosher, Pantazis, & Leahy, 2011) on MATLAB (The MathWorks Inc., 2022), largely in accordance with the pipeline described in Brainstorm tutorials (Niso et al., 2019). In the HCP data, MEG recordings were first coregistered to the subject’s structural MRI using the MEG anatomical data. We did not have access to the fiducial positions for the OMEGA dataset and thus defined approximate fiducial locations that were then refined using the digitised head points. A notch filter (60, 120, 180, 240, and 300 Hz), followed by high-pass filter (0.3 Hz) were applied to resting-state and empty-room recordings, to filter out power-supply and slow-wave/DC-offset artifacts respectively. Each subject’s recording was then visually inspected, along with the channel PSD, to weed out bad channels and bad time segments. The ECG and EOG recordings were then used to identify heartbeats and eye blinks, after which the identified artifacts were removed using their signal space projections (Uusitalo & Ilmoniemi, 1997).

Source-level activities were then estimated from sensor-level recordings at 8,004 points corresponding to the fsLR4k mesh for the HCP data and 15,002 points for the OMEGA data. This involved first computing the head model using overlapping spheres and constrained dipoles normal to the cortical surface, and estimating the noise covariance from the empty-room recordings. Source-level activities were then estimated using the dynamical statistical parametric mapping (dSPM) method (Dale et al., 2000) available in Brainstorm. The sources were then parcellated using the Schaefer-Yeo 7-network 100 atlas (*N* = 100) (Schaefer et al., 2018) for HCP and Destrieux atlas (*N* = 150) (Destrieux, Fischl, Dale, & Halgren, 2010) for OMEGA, with the parcel activity computed as the principal component of the constituent source activities.

#### Structural connectivity.

Structural brain networks for 1,000 healthy young adults from the HCP were mapped from minimally preprocessed high-resolution diffusion-weighted MRI (Glasser et al., 2013). Following previous work (Mansour L, Tian, Yeo, Cropley, & Zalesky, 2021; Seguin, Mansour L, Sporns, Zalesky, & Calamante, 2022), an MRtrix3 probabilistic tractography pipeline (Tournier, Calamante, & Connelly, 2012) was used to map whole-brain white matter tractograms—multishell multitissue constrained spherical deconvolution (Tournier, Calamante, & Connelly, 2007) iFOD2 tracking algorithm (Tournier, Calamante, & Connelly, 2010), anatomically constrained tractography (Smith, Tournier, Calamante, & Connelly, 2012), 5 M streamlines—see Mansour L et al. (2021) for further details. Cortical gray matter was parcellated using the Schaefer-Yeo 7-network 100 atlas (Schaefer et al., 2018). Connection weight between pairs of regions was defined as the number of streamlines normalized by the product of their surface areas. Connections comprising less than five streamlines were discarded to minimize the false positive rate of probabilistic tractography (Zalesky et al., 2016). A distance-dependent consensus algorithm was used to combine subject-level structural networks to a group-level structural connectome (SC) (Betzel, Griffa, Hagmann, & Mišić, 2019). A proportional threshold was then applied to the group-level SC to retain only the top 15% structural connections, the SC was binarized, and inter-hemispheric connections were removed. This SC was used for the analysis of the MEG recordings from the HCP dataset (source-localized and parcellated using the same atlas) to identify the anatomically connected neighborhood of each source.

DWI-derived subject-level SCs of 50 healthy young adults in the Destrieux parcellation (*N* = 150) were obtained from the publicly available MICA-Microstructure-Informed Connectomics (MICA-MICs) dataset (Royer et al., 2022). As done previously, we combined the subject-level SCs using a distance-dependent consensus algorithm to obtain a group-level SC, which was proportionally pruned to retain the top 15% connections. The SC was then binarized, inter-hemispheric connections were removed, and used in the analyses of the OMEGA dataset’s MEG recordings (source-localized and parcellated using the Destrieux atlas).

#### Functional connectivity.

We obtained minimally preprocessed ICA-FIX resting-state functional MRI data of the same 1,000 healthy young adults from the HCP. Four scans (two sessions: right-to-left and left-to-right phase encoding scans, on two separate days) spanning 14 min and 33 s (Repetition time, TR = 0.72 s = 0.72 s) were collected for each participant, for a total of 4,000 subject-level scans (acquisition protocols detailed in Glasser et al., 2013; Smith et al., 2013). The time series of voxels belonging to the same gray matter region delineated by the Schaefer-Yeo 7-network 100 atlas were averaged, and the FC, corresponding to each scan, was computed as Pearson correlation between all pairs of regional time series. Group-level FC was computed as the average of the 4,000 subject-level FCs (Seguin et al., 2022).

### Inferring Inter-Areal Communication

We used a previously developed method to infer communication between regions, termed EWC. EWC uses a targeted, temporally ordered, windowed approach to gauge the transmission of endogenous perturbations between neural elements from their activity time series (Madan Mohan et al., 2025). Briefly, EWC involves (a) the identification of salient features of the source region’s signal, which are termed “events”; (b) the definition of a subsample of the signal at the location of the events, and at temporally displaced locations in the target’s signal, proportional to the conduction delay between source and target; and (c) the estimation of the statistical dependence or FC between the source and target using measures such as partial correlation, conditional mutual information, and so forth. Thus, EWC effectively infers the propagation of endogenous perturbations by tracking lagged statistical relationships between source-target pairs.

The inference of communication via the EWC framework has been previously validated both in silico as well as using empirical data. In a system of four in silico nodes interacting via a simple network motif and exhibiting noise-driven dynamics and spiking (in three of the nodes to simulate spontaneous endogenous perturbations), the EWC framework using the partial correlation was shown to reliably and accurately capture asymmetric/directional interaction pathways between the sources and target under systematic variation of noise amplitudes, delays, and spiking rates. The temporal ordering that accounts for the conduction delays was also shown to diminish spurious communication between targets driven by a common source. The interactions inferred via EWC were also shown to correlate strongly with current standards like the TE (*R* ≈ 0.81) (Madan Mohan et al., 2025) while being more computationally efficient, by virtue of its targeted subsampling. For instance, MEG recordings of similar lengths used in this work were processed almost 4 times faster with EWC (measured using partial correlation) compared to conventional TE implementations (using the simplest Gaussian estimators). Additionally, the windowed approach permits the simultaneous measurement of spectral properties of the signal, allowing us to test oscillation-based communication hypotheses, making EWC ideal for this study. Moreover, since EWC is a general inference framework, it is compatible with any measure of statistical dependence to gauge communication. While partial correlation is simple and easily interpretable in time-domain analyses, it can be swapped out for other measures like the conditional mutual information, coherence, distance correlation, and so forth. depending on the use case. Notably, the temporal ordering incorporated in the EWC pipeline imparts directionality to otherwise undirected/symmetric measures.

Prior to EWC estimation, recordings were epoched into smaller 10-s segments to enable efficient computation, and each epoch was processed independently. For each epoch, event identification was done using a *z*-score based threshold (Sorrentino et al., 2021) of ∣*z* ∣ > 3.

We follow the approach and operational parameters in Madan Mohan et al. (2025), but summarize the steps below. The following steps were carried for each event:

1. A region was chosen as a source, and a communication window of 1 s was defined starting at the event. After identifying the regions that were anatomically connected to the source based on the SC matrix (neighbors), a window of similar length was placed at their time series, at delayed timepoints (delay proportional to the Euclidean distance between the source and the target). The window size was chosen to be short enough to be proximal to the event and long enough to ensure that the effects of the event would be captured, EWC estimates remained reliable, and spectral analysis could be carried out.
2. Partial correlation was used to gauge statistical dependence between the source and target recordings contained in the windows. The past activity of regions (up to one window length, 1 s) was used as the conditional variable, to discount the effects of past internal dynamics on inferred communication. When used with partial correlation, EWC has also been shown to be highly correlated with the TE and bivariate GC (Madan Mohan et al., 2025).
3. The partial correlation estimates were checked for significance, with a Bonferroni-corrected threshold of *α* = 0.01/*N*_*events*_ (*N*_*events*_ is the total number of events within the epoch), since the correlation is tested multiple times within an epoch. Estimates were set to zero above this threshold.

The EWC protocol resulted in a *N* × *N* × *M* communication matrix for a recording split into *M* epochs, for each subject.

Temporal ordering was proportional to the physical Euclidean separation between regions defined using centroid coordinates of the ROIs in the Schaefer-Yeo 7-network 100 atlas (for HCP) and the Destrieux atlas (for OMEGA) (Abeysuriya et al., 2018; Deco, Jirs, McIntosh, Sporns, & Kötter, 2009; Papadopoulos, Lynn, Battaglia, & Bassett, 2020). Inter-regional distances were proportionally converted to time delays (*δ*) as *δ_ij_* = *ED*_*ij*_/*v*, where *v* is the conduction velocity of neural signals, set to 10*m*/*s* (Papadopoulos et al., 2020). Parcellated source-localized resting-state MEG recordings were corrected for source leakage effects by removing zero-lag correlations as per Colclough, Brookes, Smith, and Woolrich (2015), using the OHBA Software Library (OSL) package in Python (Quinn, van Es, Gohil, & Woolrich, 2023).

### Neural-Oscillatory Measures

For each source-target pair of regions, the target power and phase coherence were computed alongside the EWC, for each pair of communication windows situated at the events, that is, each EWC estimate, which captured the information flow from a source to a target over a certain window of time, had an associated value of target power and phase coherence (Figure 1A).

#### Target power.

To test whether the spectral power of a target influenced how much information it received from a source, for each source-target pair, we first measured the PSD of the target signal contained within the communication window associated with each event. This was done using Welch’s periodogram method in MATLAB (function: pwelch), with a window size of *SR*/2. The power in each frequency band—theta (4–6 Hz), alpha (8–12 Hz), beta (14–24 Hz), gamma-lo (30–59 Hz), and gamma-hi (60–80 Hz)—was estimated by first integrating the PSD over the constituent frequencies through trapezoidal numerical integration, and then normalizing the estimate by the total power of the PSD to give the relative power of each band.

Similar to the output of the EWC protocol described above, for each source, we obtained a value of *N* target powers, resulting in a final *N* × *N* × *M* matrix per subject.

#### Inter-site phase clustering (ISPC).

To test whether the information transfer between a source and target was dependent upon the phase coherence between them, we measured the ISPC between the instantaneous phase time series of band-passed source and target signals contained within communication windows. ISPC, or simply the phase coherence or phase locking value, is a measure of consistency of the phase relationship between signals (Cohen, 2014; Lachaux, Rodriguez, Martinerie, & Varela, 1999), varying from 0 to 1, indicating random/independent phase relationship to perfectly consistent phase difference over time respectively. Between regions with high ISPC, the communication through coherence hypothesis (Fries, 2005) posits that information transfer should be highest for pairs regions with delay-corrected angular differences close to 0, due to their temporally coordinated excitability cycles, and lowest for those that are consistently out of phase (angular differences close to *π*).

Operating on the instantaneous phase time series, ISPC computation requires Hilbert transformation of the band-passed raw signal to create a complex-valued analytic signal. Given a pair of phase time series *φ_x_* and *φ_y_* in a frequency band *f*, ISPC is computed as:

$${\text{ISPC}}_{f}=\left|n^{-1}\sum_{t=1}^{n}e^{i\left(\varphi_{xt}-\varphi_{yt}\right)}\right|$$

where *n* is the number of time points. ISPC can alternatively be viewed as an amplitude-normalized version of the spectral coherence obtained from the Fourier transform of the cross spectral density (Bruña et al., 2018). Like EWC and power, ISPC computation between all source-target pairs resulted in an *N* × *N* × *M* matrix for each subject.

### Communication Principles

To gauge the dependence of inferred inter-areal communication on the power and phase coherence, for each subject, the Pearson correlation between each row of the EWC matrix concatenated over epochs (resulting in a 1 × *NM* vector that corresponds to a single source) and the matching epoch-concatenated row in the power and ISPC matrices was estimated. This resulted in 10 Pearson coefficients per region (five bands of power and five bands of ISPC) indicating how aligned the communication from that region (source) was to a certain communication principle. Estimating the correlation of all regions similarly yielded a *N* × 10 matrix per subject, which we term the observed “principle matrix” (Figure 1B).

### Surrogate Data Analysis

When investigating the validity of a communication mechanism by assessing the relationship between communication and the measure theorized to underpin it, it is vital to ascertain whether the measures might have some trivial or “default” dependence between them. This is particularly important in this work, since both the measure of communication and power/ISPC are derived from the same underlying MEG signal (Supporting Information Figure S1). To establish whether the observed relationship between EWC and power/ISPC significantly exceeds the trivial dependence between the measures, the elements of the *N* × 10 principle matrix capturing a subject’s regional alignment of communication to the tested spectral measures (observed correlation between EWC and power/ISPC) was compared to a cyclically permuted surrogate distribution (Figure 1C). The surrogate distribution was generated and compared to the observed principle matrix as follows:

1. We generated a surrogate dataset using a random cyclic permutation approach to shuffle the regional time series: (a) in each epoch, a random integer was chosen between 0 and the total length of the data contained in the epoch. (b) For each source region, the activities of all other regions (targets) were cyclically permuted in the forward direction by the number of steps given by the random integer. (c) The steps in the above sections were carried out on the permuted data. By cyclically permuting the signals relative to the source, we effectively compute the EWC and neural oscillatory measures between random pairs of windows—any trivial dependence between EWC and the target power or ISPC would persist between these random windows and manifest as the (surrogate) correlation between EWC and power/ISPC.
2. An *N* × 10 surrogate principle matrix is obtained for each surrogate (the ROI-level correlation between EWC and each of the neural oscillatory measures).
3. The communication protocol is carried out for *S* (= 1,000) separate surrogate datasets, resulting in a total of *S* surrogate principle matrices (each with dimensions *N* × 10) for each subject.
4. The *p* value for principle l of region i was defined as:
  $$p_{il}=\frac{\#\left(|{}_{s}^{l}R_{i}{\left|\geq \right|}_{o}^{l}R_{i}|\right)}{S}$$
  where ${}_{s}^{l}R_{i}$ and ${}_{o}^{l}R_{i}$ represent region *i*‘s surrogate and observed Pearson correlation coefficients respectively for principle *l*, and #(…) denotes the number of instances the argument is true. If the observed dependence between EWC and the neural oscillatory measures simply reflects the trivial dependence between the measures, the *p* value will not pass the significance threshold.
5. The *p* values were false discovery rate (FDR) corrected across rows using the Benjamini-Hochberg linear step-up procedure implemented in MATLAB (function “mafdr”).
6. A subject-level mask with dimensions *N* × 10 was defined, such that *mask*_*il*_ = 1 if *p*_*il*_ < 0.05. This mask was applied to the observed *N* × 10 principle matrix of the subject.

The surrogate-corrected *N* × 10 subject-level principle matrices were averaged across subjects and projected to the cortical surface (Figure 1B).

### Functional Gradient Analysis

Functional gradients were computed through dimensionality reduction of group-level FC (derived from resting state functional magnetic resonance imaging) from the HCP, using diffusion embedding. We used the principal gradient or eigenvector of the diffusion operator, which varies across the unimodal to transmodal axis. The sign of the obtained gradients were flipped to enable comparison to existing works (Margulies et al., 2016). This method was implemented using the Dimensionality Reduction Toolbox (Van Der Maaten, Postma, & Van Den Herik, 2009).

To establish whether the alignment to the tested measures varied based on where a region was situated on the unimodal-transmodal gradient, we estimated the Spearman correlation strength between the principal gradient values and the EWC-power/ISPC alignment (elements of the surrogate corrected principle matrix).

### Statistical Analyses

Correlations between EWC and neural oscillatory measures for each region were tested for statistical significance using a cyclic-surrogate-based permutation approach (see Surrogate Data Analysis. For each subject (*n* = 30 for the HCP dataset and *n* = 5 for the OMEGA dataset), we created a surrogate dataset with *S* = 10^3^ surrogates. Each cyclic surrogate involved cyclically shifting the MEG recording data of targets by a random amount with respect to each source region’s recording, after which correlations between EWC and neural oscillatory measures were estimated. A *p* value was defined by comparing each surrogate correlation strength with the observed as follows: $p_{il}=\#\left(|{}_{s}^{l}R_{i}{\left|\geq \right|}_{o}^{l}R_{i}|\right)/S$, for a region *i*, and neural oscillatory principle *l*. To account for multiple comparisons, the *p* values were then FDR corrected using the Benjamini-Hochberg linear step-up procedure across regions (*N* = 100 for HCP and = 150 for OMEGA). The correlation values were then thresholded based on the FDR-corrected *p* values (*α* = 0.05), and set to 0 if non-significant (indicating no relationship between EWC and the oscillatory measures).

To identify the top oscillatory principle for each region, we picked out the oscillatory measure that maximally correlated with each ROI’s information outflow (Figure 2D, top and Figure 3D, top). To ensure that the top oscillatory principle explained communication significantly better than the other tested measures, we performed a one-way ANOVA across correlation values (separately for target power and ISPC). For the ROIs in which the ANOVA indicated a significant difference between the alignment to different mechanisms, we carried out pairwise comparisons between all group means (multiple comparison test using Tukey–Kramer honestly significant difference procedure (Tukey, 1949) and assessed the significance of the mean difference between the top two communication principles (*α* = 0.05) (Figure 2D, middle, bottom; Figure 3D, middle, bottom). Pairwise comparisons using the same procedure were also carried out between mean correlation across regions (Figures 2C and 3C). The significance of the relationship between EWC-power/ISPC correlations and functional hierarchy along the unimodal-transmodal axis was tested through spin-permutation (*s* = 10^4^ permutations) of the parcellated cortical maps using the technique employed in (Váša et al., 2018).

## ACKNOWLEDGMENTS

V.M.M. was supported by the Melbourne Research Scholarship, University of Melbourne. A.M.H. was supported by the Australian Research Council (DE220101019). C.S. acknowledges support from the Australian Research Council (DP170101815). A.Z. is supported by an ARC Future Fellowship (FT220100091) and the Rebecca L. Cooper Foundation. R.F.H.C. is funded by an National Health and Medical Research Council Emerging Leadership Investigator Grant (2017527). Data were provided (in part) by the Human Connectome Project, WU-Minn Consortium (Principal Investigators: David Van Essen and Kamil Ugurbil; 1U54MH091657) funded by the 16 National Institutes of Health (NIH) Institutes and Centers that support the NIH Blueprint for Neuroscience Research; and by the McDonnell Center for Systems Neuroscience at Washington University. This research was supported by The University of Melbourne’s Research Computing Services and the Petascale Campus Initiative.

## SUPPORTING INFORMATION

Supporting information figures: Figure S1, Figure S2, Figure S3. Supporting information for this article is available at https://doi.org/10.1162/NETN.a.550.

## AUTHOR CONTRIBUTIONS

Varun Madan Mohan: Conceptualization; Formal analysis; Investigation; Methodology; Software; Validation; Visualization; Writing – original draft. Thomas F. Varley: Conceptualization; Methodology; Validation; Writing – review & editing. Anthony M. Harris: Validation; Writing – review & editing. Robin F. H. Cash: Validation; Writing – review & editing. Caio Seguin: Conceptualization; Investigation; Methodology; Supervision; Validation; Writing – review & editing. Andrew Zalesky: Conceptualization; Investigation; Methodology; Project administration; Supervision; Validation; Writing – review & editing.

## FUNDING INFORMATION

Anthony M. Harris, Australian Research Council (https://dx.doi.org/10.13039/501100000923), Award ID: DE220101019. Caio Seguin, Australian Research Council (https://dx.doi.org/10.13039/501100000923), Award ID: DP170101815. Andrew Zalesky, Australian Research Council (https://dx.doi.org/10.13039/501100000923), Award ID: FT220100091. Varun Madan Mohan, Melbourne Research, University of Melbourne (https://dx.doi.org/10.13039/501100000987). Andrew Zalesky, Rebecca L. Cooper Medical Research Foundation, (https://dx.doi.org/10.13039/501100001061). Robin F. H. Cash, National Health and Medical Research Council (https://dx.doi.org/10.13039/501100000925).

## DATA AND CODE AVAILABILITY

All the code used to analyze data is available at https://github.com/vmadanmohan/flex-comm-principles.

## Supplementary Material



## TECHNICAL TERMS

Power: Magnitude of the various frequency components that make up a signal/recording associated with a region.
Event-marked Windowed Communication (EWC): An estimate of communication between brain regions, inferred from altered functional connectivity as information flows between them.
Source: A region from which information flow originates, capable of altering the activity of other regions.
Target: A region that can potentially receive information from a source.
Inter-site phase clustering (ISPC): A measure of how consistent the phase difference between signals/recordings of a pair of regions is, across time.
